# Size of Metastatic Lymph Nodes

**DOI:** 10.1155/2013/648219

**Published:** 2013-04-11

**Authors:** L. Uriev, I. Maslovsky, F. Barak, D. Ben-Dor

**Affiliations:** ^1^Institute of Pathology, Barzilai Medical Centre, 7830604 Ashkelon, Israel; ^2^Tel Hai Clinic, 7751056 Ashdod, Israel; ^3^Institute of Oncology, Barzilai Medical Centre, 7830604 Ashkelon, Israel

## Abstract

We present a case and review of the literature of well-differentiated sigmoid adenocarcinoma with numerous metastases into pericolic lymph nodes. All positive lymph nodes were small. The authors concluded that there is no clear correlation between nodal size and the likelihood of metastasis in the lymph node, and the status of small lymph nodes must receive special attention by clinicians and pathologists.

## 1. Case Report

A 64-year-old man presented with rectal bleeding and anemia. Colonoscopy revealed polypoid tumor in sigmoid colon. The biopsy showed well-differentiated adenocarcinoma. Abdominal computerized tomography demonstrated neither regional nor distant metastasis. Anterior rectosigmoid resection was performed. The postoperative course was unremarkable.

## 2. Pathological Findings

**Figure 1:**
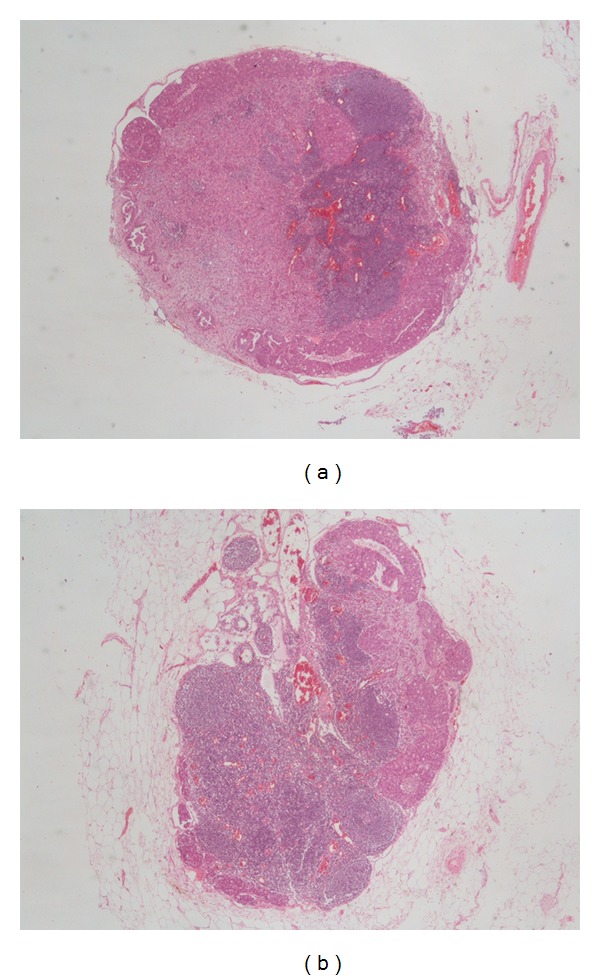
Small metastatic lymph nodes (haematoxylin and eosin stain, ×20).

Large bowel fragment 12 cm in length showed a 4 cm polypoid tumor of sigmoid colon. Microscopic examination revealed well-differentiated adenocarcinoma arising in preexisting tubulovillous adenoma and invading into submucosa with foci of vascular invasion. Metastases were found in 12 of 13 pericolic lymph nodes. Diameter of metastatic nodes was from 1.5 mm up to 5 mm with partial to subtotal replacement of lymph node parenchyma and focal extracapsular extension. There were 8 involved lymph nodes from 1.5 mm to 2.9 mm in diameter. And 4 metastatic nodes had diameter from 3.0 mm to 5 mm. The mean size of involved lymph nodes was 2.9 mm (Figures 1(a) and 1(b)).

## 3. Discussion and Review

The standard assessment of nodal status requires a histological examination of the lymph nodes recovered from the mesocolic or perirectal tissues. The number of involved lymph nodes is a relevant prognostic parameter which determines the duration of survival in patients with colonic carcinoma. Does the nodal size reflect the likelihood of metastasis in the lymph node? The general point of view is that there is positive correlation between the above. But in our case all involved nodes were small (most of them were less or equal to 2.9 mm) and were not found at computerized tomography examination of abdominal cavity before the operation. Different opinions are present in the literature.

So, Cserni concluded that metastatic lymph nodes are significantly larger than uninvolved ones. Positive nodes tend to be larger, but reactive ones may also be large. The size has much to do with the detectability of a lymph node; large nodes are easier to recover [1].

Kotanagi et al. found only a nonsignificant trend for positive nodes to be larger than negative ones [2].

In contrast, Mönig et al. reported that metastatic nodes were on the whole larger [3].

Bjelovic et al. started that within the group of small lymph nodes, 17% were malignant. Additionally, of all the malignant lymph nodes, 46% were less than 5 mm in diameter. Small lymph nodes are commonly nonpalpable. Size and consistency of lymph nodes are not dependable parameters for appraisal of lymph node involvement in tumor tissue [4].

Regarding the other tumoral locations in the body, no clear correlation of lymph node size and metastatic involvement is seen. For example, Vogel et al. measured the diameter of hilar and mediastinal lymph nodes in bronchial cancer. They found no sufficient correlation between the diameter of the lymph node and their infiltration by cancer cells [5].

Prenzel et al. noted that preoperative lymph node staging of lung cancer by computerized tomography relied on the premise that malignant lymph nodes were larger than benign ones. Frequency of metastatic involvement was calculated and correlated with lymph node size. The conclusion was that lymph node size was not a reliable parameter for the evaluation of metastatic involvement in patients with nonsmall cell lung cancer [6].

Macdonald et al. explored the level VI node size as a predictor of malignancy in papillary thyroid cancer. They concluded that the decision to perform a level VI neck dissection could not be based on a preoperative ultrasound size [7].

On the other hand, lymph nodes measuring larger than or equal to 4 mm, especially those located anterior to the midportion of the aorta, should raise a suspicion of metastases in patients with clinical stage I testicular nonseminomatous germ cell cancer [8].

In summary, we can see that the results of different studies are contradictory, because there is no clear correlation between nodal size and the likelihood of the metastasis in lymph node. In our case, the correlation is definitely negative. Although preoperative clinical lymph node staging relies on the supposition that malignant lymph nodes are larger than benign ones, the metastatic status of the small lymph nodes must receive special attention not only by clinicians, but also by pathologists who should aim to recover as many lymph nodes as possible.

## 4. Take Home Messages

- For clinicians: small lymph nodes can be metastatic, and large ones can be reactive.
- For pathologists: small lymph nodes should not be neglected and must be picked up and examined thoroughly.
